# Relationship of oxidative stress and antioxidant response with vaso-occlusive crisis in sickle cell anaemia

**DOI:** 10.4314/ahs.v21i1.20

**Published:** 2021-03

**Authors:** Godwill Azeh Engwa, Amanda Okolie, John Paul Chinedu Chidili, Precious Amara Okore, Paul Chigozie Onu, Maryrose Onyinye Ugwu, Daniella Ebeshe Oko, Paschaline U Ferdinand

**Affiliations:** 1 Biochemistry, Department of Chemical Sciences, Godfrey Okoye University, P.M.B 01014, Thinkers Corner, Enugu Nigeria; 2 Department of Biological and Environmental Sciences, Faculty of Sciences, Walter Sisulu University, Mthatha 5117, South Africa; 3 Biotechnology, Department of Biological Sciences, Godfrey Okoye University, P.M.B 01014, Thinkers Corner, Enugu Nigeria; 4 Bio-resources Development Center Arochukwu, National Biotechnology Development Agency (NABDA), Abuja Nigeria

**Keywords:** Sickle cell anaemia, oxidative stress, antioxidant, vaso-occlusive crisis

## Abstract

**Background:**

Though sickle cell anaemia (SCA) is known to promote oxidative stress, there is paucity of information on the relationship between oxidative stress and vaso-occlusive crisis (VOC).

**Objective:**

This study was undertaken to evaluate the relationship of oxidative stress and antioxidant response with VOC in SCA.

**Methods:**

A cross-sectional case-control study was carried out at University of Nigeria Teaching Hospital (UNTH), Ituku-Ozalla, Enugu Nigeria involving 116 individuals which included 36 SCA subject, 40 sickle cell carriers (AS) and 40 healthy individuals (AA). Baseline information as well as the frequency of VOC was obtained from the participants and anaemia as well as oxidative stress and antioxidant indices were assessed in blood.

**Results:**

Anaemia was prevalent (88.9 %) in SCA individuals compared to AS (52.5%) and AA (47.5 %) individuals. Nitric oxide scavenging (NOS) and superoxide dismutase (SOD) activities as well as glutathione level were significantly (p<0.005) lower while catalase activity was higher in SCA individuals compared to controls (AA and AS). Higher malondialdehyde (MDA) level was associated with very severe VOC while low level of NOS activity was associated with severe VOC in SCA individuals.

**Conclusion:**

Sickle cell anaemia exhibited oxidative stress and alteration in the levels of antioxidant indices which was possibly associated with vaso-occlusive crisis.

## Introduction

Sickle cell anaemia (SCA) is an inherited blood disorder which is of major health concern especially in Africa, Southeast Asia and the Americas. Globally, about 5% of the population is affected by some type of haemoglobin variants, and over 300,000 children are born each year with haemoglobinopathies, with SCA being the most prevalent type^1^, ^2^. It is estimated that the prevalence of live births with the disease is 4.4% in the world with higher rates in Africa, Southeast Asia and the Americas. Sickle cell trait prevalence ranges from 10 to 45% in most parts of sub-Saharan Africa^3^,^4^,^5^. In Nigeria, the prevalence of sickle cell carriers ranges between 20 and 30% while SCA affects about 2 to 3% of Nigerians translating to about 3 million cases^6^.

SCA results from a point mutation in the β-globin chain due to the substitution of Valine for Glutamine at position 6, shifting the isoelectric point of the protein^7^. This mutation causes red blood cells (RBCs) to lose their disc shape along with their elasticity due to a low oxygen tension and assumes an inflexible crescent shape forming a sickled RBC. This sickle shaped RBC results in vaso-occlusion, which is the obstruction of blood vessels as a result of the inability of the sickled RBCs to bend and twist as they move through the narrow blood vessels^8^. The actual cause of anaemia in this illness is due to haemolysis which results from the breakdown of RBCs, because of their sickled shape. Clinical manifestations of SCA usually begin in childhood and may lead to various acute and chronic complications. The severity, frequency and duration of these complications vary from person to person and are potentially lethal. One of such complications is the vaso-occlusive crisis (VOC), a condition caused by the accumulation of sickle-shaped RBCs in capillaries obstructing and restricting blood flow into organs, thereby resulting in pain, ischaemia and often leads to organ damage^9^. This blockage of capillaries induces the recruitment of neutrophils and monocytes thereby promoting chronic pro-inflammatory response^10^. Inflammatory responses are usually accompanied by the action of reactive oxygen species (ROS) and thus, there is a possibility that VOC may be influenced by oxidative stress.

Oxidative stress is a condition whereby ROS overwhelms the antioxidant defence system. In SCA, there is recurrent polymerization of HbS and depolymerization which leads to the autoxidation of HbS and increased metabolic turnover that result in increased generation of ROS. As such, sickle erythrocytes may be a source of prooxidants in SCA^11^. Other major sources of ROS are thought to be the increased xanthine oxidase activity in sickle cell disease aortic endothelium and increased level of leucocytes, which doubles the number of superoxide production in sickle cell disease^12^. Also, sickle erythrocytes have been reported to generate twice the number of superoxide (O^2^•−), hydrogen peroxide (H_2_O_2_), hydroxyl radical (HO•) as well as lipid oxidation products compared to the HbA erythrocytes^11^. Usually, there are antioxidants; both enzymatic and non-enzymatic molecules that play a crucial role in maintaining the balance of ROS in the body.

Antioxidants are molecules that quench or inhibit the actions of free radical as well as prevent cellular damage. Antioxidants exist as both enzymatic and non-enzymatic molecules in the body 13. Enzymatic antioxidants act by metabolising free radicals and removing them from cells. Most of these antioxidant enzymes convert reactive oxidative species to H_2_O_2_ and subsequently to water, in the presence of cofactors such as manganese, iron, zinc, and copper in a multi-step process. Non-enzymatic antioxidants act by interfering or interrupting chain reactions of free radicals. Major defence mechanisms against ROS include superoxide dismustase (SOD), catalase, glutathione peroxidase (GPx) as well as non-enzymatic molecules including reduced glutathione (GSH), uric acid, ubiquinols, flavonoids, carotenoids, vitamins C and E^14^.

In overwhelming levels of ROS over the antioxidant system, there is high level of free ROS leading to a state of oxidative stress. Thus, oxidative stress is described as an imbalance between oxidants or free radicals and antioxidants^15^,^16^. This imbalance may lead to attack on all the cell components by ROS thereby damaging cells and leading to diseases^15^. Oxidative stress can damage specific molecular targets such as proteins, lipids, nucleotides etc, resulting in cell dysfunction and/or death. There is an indication that increased reactive radical species are linked with increased oxidation in sickle cell individuals which are highly damaging to the cells^17^,^18^. Though, there exist evidences suggesting that oxidative stress is responsible for increased pathophysiology of secondary dysfunctions in sickle cell patients^19^, there is paucity of information on the effect of oxidative stress on vaso-occlusive crisis in sickle cell anaemia. Thus, this study was undertaken to evaluate the relationship of oxidative stress and antioxidant response on vaso-occlusive crisis in sickle cell anaemia.

## Methods

### Study population and design

This was a cross-sectional case-control study carried out at the University of Nigeria Teaching Hospital (UNTH), Ituku-Ozalla, Enugu Nigeria. Ethical approval was obtained from the institution review board of UNTH with approval number: UNTH/CSA/329/OL.5. Recruitment of participants was done in accordance with the Helsinki Declaration (revised version 2008) whereby written informed consent was obtained from participants before enrolment into the study. Participants were randomly recruited which involved SCA individuals of the SS genotype while individuals with genotypes AA and AS genotypes served as controls.

### Inclusion/exclusion criteria

Individuals with/or without SCA were recruited for the study. Pregnant and lactating mothers as well as individuals with obesity, metabolic syndrome, cancer, peripheral vascular disease, autoimmune diseases were excluded from the study.

### Data and blood collection

A questionnaire was used to obtain baseline information of participants including age, sex, genotype, state of origin and the frequency of vaso-occlusive crisis characterized by sudden onset of excruciating pain. The individual's systolic and diastolic blood pressure was measured using an automatic blood pressure monitor. A volume of 5ml of blood was collected into an anticoagulant tube (EDTA tube) and properly swirled to avoid clotting.

### Determination of anaemia indices

Anaemia indices including haemoglobin concentration (Hb) and pack cell volume (PCV) were assayed using an automatic Hb Haemoglobin testing system.

### Assessment of oxidative stress and antioxidants

The method of Niehaus and Samuelsson was adopted for the determination of lipid peroxidation in blood with slight modifications^20^. The protein carbonyl content (PCC) was quantified according to the method of Mesquita and colleagues^21^. Nitric oxide scavenging (NOS) activity was determined as per the method of Garrat^22^. The reduced glutathione (GSH) content of the blood sample was estimated according to the method described by Sedlak and Lindsay^23^. Superoxide dismutase (SOD) activity was determined according to the method of Sun and Zigma^24^. Determination of glutathione-S-transferase (GST) was based on the fact that GST demonstrate a relatively high activity with 1-chloro-2, 4-dinitrobenzene which is subsequently conjugated with reduced glutathione^25^. The method described by Pari and Latha was adopted for the determination of catalase (CAT) activity^26^.

### Data classification

Participants were classified as anaemic when the Hb was < 12 mg/dl for women and 13 mg/dl for men according to the WHO classification. Anaemia was further classified as mild, moderate, and severe anaemia with Hb between 10.0 and 10.9 g/dL, 7.0 and 9.9 g/dL and less than 7.0 g/dL respectively. Vaso-occlusive crisis was classified as moderate when the frequency of the crisis was <3 times/year, severe when it was between 4–6 times/year and very severe when it was >6/years. Antioxidant and oxidative stress indices were classified as follows: CAT (U/mg) as Low: <2000 and High: ≥ 2000; SOD (U/mg) as Low: <500 and high: ≥ 500; GST (U/ml) as Low: <6 and High: ≥ 6; GSH (µg/ml) as Low: <300 and High: ≥ 300; NOS (%) Low: <200 and High: ≥ 200; MDA (µM) as Low: <8 and High: ≥ 8; PCC (µM) as Low: <80 and High: ≥ 80.

### Statistical analysis

Statistical Package for Social Sciences (SPSS) version 23 was used for data analyses and the data was expressed as mean ± standard error of the mean (S.E.M). Comparison of continuous variables for various groups (AA, AS, SS) was done using analysis of variance (ANOVA). The proportions of categorical data (anaemia, sex, age group and VOC) were compared for the various groups using chi-square. A 95% confidence interval was considered and a difference was significant at p-value ≤ 0.05.

## Results

### Baseline characteristics of subjects

A total of 116 subjects were recruited for the study which included 40 individuals of AA genotype, 40 individuals of AS genotype, and 36 of SS genotype. Among the 116 individuals, there were 50 males amongst which 21(42.0%) were AA, 8(16.0%) were AS and 21(42.0%) were SS while there were 66 females of which 19(28.8%) were AA, 32(48.5%) were AS and 15(22.7%) were SS. The ages of the study participants were insignificantly different (p = 0.162) among the various genotypes. The systolic and diastolic blood pressures were significantly (p = 0.05) lower in subjects with SS genotype. Results are summarized in Table 1.

**Table 1:** Baseline characteristics of study participants

Parameters	AA (%)	AS (%)	SS (%)	Total (%)	f / χ^2^	*p*-value
**Sex**						
**Male**	21 (42.0)	8 (16.0)	21 (42.0)	50 (100.00)	13.551	0.001
**Female**	19 (28.8)	32 (48.5)	15 (22.7)	66 (100.00)		
**Age**						
**0–17**	3 (100.0)	0 (0.0)	0 (0.0)	3 (100.0)	11.766	0.162
**18–30**	32 (31.4)	36 (35.3)	34 (33.3)	102 (100.0)		
**31–45**	2 (28.6)	4 (57.1)	1 (14.3)	7 (100.0)		
**46–65**	3 (75.0)	0 (0.0)	1 (25.0)	3 (100.0)		
**State of** **Origin**						
**Enugu**	16 (23.2)	30 (43.5)	23 (33.3)	69 (100.0)	31.062	0.028
**Anambra**	8 (44.4)	1 (5.6)	9 (50.0)	18 (100.0)		
**Imo**	5 (50.0)	3 (30.0)	2 (20.0)	10 (100.0)		
**Cross**	4 (100.0)	0 (0.0)	0 (0.0)	4 (100.0)		
**Rivers**						
**Rivers**	2 (50.0)	1 (25.0)	1 (25.0)	4 (100.0)		
**Plateau**	1 (50.0)	1 (50.0)	0 (0.0)	2 (100.0)		
**Abia**	4 (80.0)	1 (20.0)	0 (0.0)	5 (100.0)		
**Ebonyi**	0 (0.0)	1 (50.0)	1 (50.0)	2 (100.0)		
**Benue**	0 (0.0)	1 (100.0)	0 (0.0)	1 (100.0)		
**Delta**	0 (0.0)	1 (100.0)	0 (0.0)	1 (100.0)		
**BP**						
**SBP**	123.28±2.35	117.68±3.64	104.39±4.32	115.48±2.12	7.538	0.001
**DBP**	73.45±1.36	74.78±2.50	58.61±2.98	69.30±1.49	14.249	<0.001

BP: Blood pressure; SBP: Systolic blood pressure; DBP: Diastolic blood pressure

### Anaemia status of subjects

The prevalence of anaemia was greatest in participants with the SS genotype totaling 88.9 % (32/36), followed by AS participants with 52.5% (21/40) and AA participants with 47.5 % (19/40). The severity of anaemia varied among participants of the various genotypes. Severe anaemia was significantly higher (p<0.001) in sickle cell subjects (SS) compared to AA and AS subjects (Table 2).

**Table 2:** Proportion of anaemic subjects in the study

Anaemia Level	Genotype			Chi- Square	*P*-value
		
	AA (%)	AS (%)	SS (%)		
**Mild**	13(68.4)	4(19.0)	2(6.2)		
**Moderate**	6(31.6)	17(81.0)	5(15.6)	**63.586**	**<0.001**
**Severe**	0(0.0)	0(0.0)	25(78.1)		
**Total**	19(100)	21(100)	32(100)		

### Oxidative stress and antioxidant indices

The level of Malondialdehyde was significantly higher (p=0.013) in AS than in AA and SS participants but the difference between AA and SS participants was insignificant (p=0.550). More so, the protein carbonyl content was insignificantly (p=0.195) higher in SS participants compared to AA and AS participants. The nitric oxide scavenging (NOS) capacity was significantly lower (p<0.05) in SS compared to AA and AS participants. A significant decreased (p<0.05) NOS activity was observed between AA and SS participants. Catalase activity was significantly higher (p<0.001) in sickle cell anaemia (SS) participants compared to AA and AS participants. SOD activity was significantly lower (p = 0.014) in sickle cell anaemia (SS) participants compared to AA and AS participants. Similarly, glutathione level was significantly lower (p<0.001) in sickle cell anaemia participants compared to AA and AS participants. Glutathione-s-transferase activity was insignificantly higher (p = 0.343) in sickle cell anaemia participants compared to AA and AS participants (Table 3).

**Table 3:** Oxidative stress and antioxidant indices in study subjects

	AA	AS	SS	*p*-value
MDA (µM)	7.59±0.46	9.00±0.35^a^	7.89±0.15^b^	0.013
Protein oxidation (µM)	64.16±2.03	59.16±2.21	65.58±3.60	0.195
NOS Activity (%)	210.87±6.03	197.60±3.10	186.01±7.2^8^a	0.010
Catalase (U/mg)	2677.28±118.38	1607.63±195.75^a^	2742.26±249.75^b^	<0.001
SOD (U/mg)	917.98±134.81	728.16±134.25	402.83±75.13^a^	0.014
GSH(µ/mg)	1759.10±120.61	1356.82±138.95^a^	555.33±107.41^ab^	<0.001
GST (U/ml)	4.37±0.99	3.57±0.86	5.45±0.79	0.343

**Legend:** AA: Normal individuals with AA genotype; AS: Sickle cell carrier; SS: Sickle cell anaemia participants; MDA: Malondialdehyde; NOS: nitric oxide scavenging activity; SOD: Superoxide dismutase; GSH: reduced Glutathione; GST: Glutathione-S-transferase.

### Relationship of vaso-occlusive crisis with oxidative stress and antioxidant indices

There was a significant (p<0.05) higher level of very severe vaso-occlusive crisis in sickle cell anaemia subjects with high MDA level compared to their counterparts with low MDA level. Also, SCA subjects with low level of NOS activity had more significant (p<0.05) severe vaso-occlusive crisis than SCA individual with high NOS activity (Table 4).

**Table 4:** Relationship of vaso-occlusive crisis with oxidative stress and antioxidant indices

	Painful vaso-occusion crisis						

		Mild (%)	Severe	Very Severe	Total	Chi-square	*p*-value
**CAT(U/mg)**	**Low**	11 (31.4)	7 (20.0)	2 (5.7)	20 (57.1)	0.173	0.917
	**High**	8 (22.9)	6 (17.1)	1 (2.9)	15 (42.9)		
**SOD(U/mg)**	**Low**	12 (34.3)	7 (20.0)	3 (8.6)	22 (62.9)	2.226	0.329
	**High**	7 (20.0)	6 (17.1)	0 (0.0)	13 (37.1)		
**GST(U/ml)**	**Low**	13 (37.1)	10 (28.6)	2 (5.7)	25 (71.4)	0.310	0.856
	**High**	6 (17.1)	3 (8.6)	1 (2.9)	10 (28.6)		
**GSH(µ/mg)**	**Low**	14 (40.0)	10 (28.6)	1 (2.9)	25 (71.4)	2.373	0.305
	**High**	5 (14.3)	3 (8.6)	2 (5.7)	10 (28.6)		
**NOS (%)**	**Low**	9 (25.7)	12 (34.3)	2 (5.7)	23 (65.7)	6.920	0.031
	**High**	10 (28.6)	1 (2.9)	1 (2.9)	12 (34.3)		
**MDA(µM)**	**Low**	14 (40.0)	8 (22.9)	0 (0.0)	22 (62.9)	6.041	0.049
	**High**	5 (14.3)	5 (14.3)	3 (8.6)	13 (37.1)		
**PCC(µM)**	**Low**	16 (45.7)	9 (25.7)	2 (5.7)	27 (77.1)	1.187	1.553
	**High**	3 (8.6)	4 (11.4)	1 (2.9)	8 (22.9)		

PCC: Protein carbonyl content; CAT: Catalase activity; GST: Glutathione-S-Transferase activity; GSH: Glutathione; SOD: Superoxide dismutase activity; NOS: Nitric oxide scavenging activity; MDA: Malondialdehyde.

## Discussion

In SCA, anaemia can be caused by blood loss, decreased red blood cell production, and increased red blood cell breakdown. In this study, it was observed that the prevalence of anaemia, particularly severe anaemia was significantly higher in SCA individuals compared to controls. This finding is similar to that of a previous report among sickle cell patients in Nigeria^27^. SCA is known to generate ROS during the auto-oxidation of HbS which is associated with reduced antioxidant defence mechanism^28^. Apart from HbS autoxidation, other intrinsic source of pro-oxidant production included heme iron release, increased activity of certain oxidases such as endothelial xanthine oxidase and NADPH oxidase^29^, uncoupling of NOS activity and decreased nitric oxide (NO) levels^30^. Elevated levels of ROS in the body beyond the antioxidant system capacity lead to a state of oxidative stress. One of the major consequences of oxidative stress is cell damage which is as a result of ROS attacking lipid membrane through the process of lipid peroxidation. One of the key products of lipid peroxidation is malondialdehyde (MDA) and has usually been considered as a key marker of oxidative stress^31^. Previous studies have shown elevated level of MDA in SCA suggesting the excessive formation of free radicals by sickle cells 32. In this study, MDA level was significantly different in SCA participants compared to AA and SS participants. Endogenous proteins are targets for free radical attacks via the oxidation of cysteine, methionine, and/or tyrosine residues; forming carbonyls as the oxidation product leading to protein damage. As such, there is a direct link between the generation of free radicals, possibly from sickle RBC and increased levels of protein carbonyls^33^. In this study, there was a trend of increased protein carbonyl content in SCA participants suggesting possible protein damage by ROS as a result of SCA. The pathophysiology of SCA is highly affected by the oxidant/antioxidant status^34^. SOD is one of the most effective intracellular enzymatic antioxidants and it catalyzes the conversion of superoxide anions to oxygen and H_2_O_2_. In this study, SOD activity was significantly lower in SCA individuals compared to the controls. This finding is in accordance with some previous studies which have shown decreased SOD in sickle cell disease^35^, ^36^. It is suggested that this decrease could be due to increased oxidative stress which results in excessive antioxidant consumption leading to antioxidant deficiency^35^ or related to the severity of the SCA disease. Catalase is an antioxidant which can decompose H_2_O_2_ to water and oxygen in reactions. There has been discrepancy in reports on catalase activity in SCD as some studies have shown decreased catalase activity in transgenic sickle mouse models^37^ as well as in SCD patients^39^ while other studies have shown increased catalase activity^38^. In this study, catalase showed increased activity in SCA individuals compared to controls. This result corroborates with findings of previous studies which showed increased catalase levels in SCD patients^38^. The increase in catalase activity might be as a result of a protective measure by the body to scavenge ROS, particularly H_2_O_2_ or might possibility be a consequence of higher reticulocyte content in SCD patients' blood, whereas the decreased levels might be due to the overwhelming and chronic level of oxidative stress^34^. NO is an important regulator of blood flow, vascular tone, and adhesion. However, in SCD, NO was found to decrease as it reacts with high level of ROS such as peroxide, superoxide, and hydroxyl radicals, forming more potent reactive NO species which may damage the cell membrane^44^. In this study, NO scavenging activity was significantly higher in SCA individuals compared to normal individuals with AA genotype. The high level of NO may serve as a protective mechanism to remove the more potent reactive NO species form in sickle cell erythrocytes. Glutathione is an antioxidant that acts as a co-factor for several detoxifying enzymes, scavenge singlet oxygen and hydroxyl radical directly, participate in the transportation of amino acid across plasma membrane, and assist in the generation of vitamins E and C^41^,^42^. In this study, glutathione activity was significantly lower in SCA individuals compared to the control groups. This finding is in accordance with previous works which have also shown glutathione level to be significantly reduced in SCD patients^43^ with some studies presenting about 50% decrease of glutathione level in sickle erythrocytes compared with normal erythrocytes^44^. Glutathione-S-transferase is an antioxidant which plays a significant role in protecting against oxidative stress by conjugating ROS with glutathione^45^. This study showed glutathione-S-transferase activity to be insignificantly higher in SCA individuals compared to the control groups. The insignificantly high level of glutathione-s-transferase may account for the lower level of glutathione in SCA individuals as it conjugates glutathione to the excess ROS formed by sickle cell erythrocytes.

SCA patients usually experience a painful episode called sickle cell vaso-occlusive crisis (VOC). This painful episode is often characterized by severe pain in the chest, back, abdomen or extremities. Usually, multiple areas of the body are affected and these painful episodes last for days or weeks. The severity, frequency and duration of this pain vary considerably amongst SCA individuals. Though it remains unclear how this pain originates, it is believed to result from the accumulation of sickle-shaped red blood cells in capillaries obstructing and restricting blood flow into organs thereby causing pain, ischaemia and organ damage^9^. Moreover, this obstructive capillary blockage induces the recruitment of neutrophils and monocytes thereby promoting chronic pro-inflammatory response^10^. Reactive oxygen species (ROS) are often released and present in chronic inflammation thus, there is a possibility that oxidative stress may influence VOC observed in SCA individuals. The disruption of the antioxidant defence system due to oxidative stress prompted us to investigate their relationship with VOC. Our findings showed a significantly higher level of very severe VOC observed in SCA individuals with high MDA level. More so, SCA individuals with low level of NOS activity had more significantly severe VOC. These findings suggest that VOC in SCA may be associated with oxidative stress. However, a limitation in this study may be the small sample size which may be limiting to the generalization of this finding.

## Conclusion

SCA exhibited oxidative stress and alteration of the antioxidant defence system which may be associated with vaso-occlusive crisis. This is the first report to present the possible association of vaso-occlusive crisis with oxidative stress in SCA subjects. However, limitation in this study was the small sample size which may be limiting to generalization. As such, further studies are required with larger sample size to ascertain the relationship between vaso-occlusive crisis in SCA individuals with oxidative stress.

## References

[1] World Health Organization Sickle-cell disease and other hemoglobin disorder; Media Centre: Who Technical Report Series, Fact sheet 2011.

[2] Modell B, Darlison M (2008). Global Epidemiology of Hemoglobin Disorders and Derived Service Indicators. Public Hlth Rev.

[3] WHO (2013). Sickle cell disease prevention and control. Regional office for Africa.

[4] Okwi AL, Byarugaba W, Ndugwa CM, Parkes A, Ocaido M, Tumwine JK (2010). An up-date on the prevalence of sickle cell trait in Eastern and Western Uganda. BMC Blood Disorders.

[5] Serjeant GR, Serjeant BE (2001). The epidemiology of sickle cell disorder: a challenge for Africa. Arch. Ibadan Med.

[6] Uzoegwu PN, Onwurah AE (2003). Prevalence of haemoglobinopathy and malaria diseases in the population of old Aguata Division, Anambra State, Nigeria. Biochemistry.

[7] Stuart MJ, Nagel RL (2004). Sickle-cell disease. Lancet.

[8] Jones P (2008). Gene and Disease: Sickle Cell Disease.

[9] Obeagu EI, Ochei KC, Nwachukwu BN, Nchuma BO (2015). Sickle Cell Anaemia: A review. Scholars J Appl Med Sci.

[10] Queiroz RF, Lima ES (2013). Oxidative stress in sickle cell disease. Rev Bras Hematol Hemoter.

[11] Silva DGH, Juniora EB, De Almeidab EA, Bonini-Domingosan CR (2013). Oxidative stress in sickle cell disease: An overview of erythrocyte redox metabolism and current antioxidant therapeutic strategies. Free Radical Biol Med.

[12] Wood KC, Granger DN (2007). Sickle cell disease: Role of reactive oxygen and nitrogen metabolites. Clin Exp Pharmacol Physiol.

[13] Valko M, Leibfritz D, Moncol J, Cronin M, Mazur M, Telser J (2007). Free radicals and antioxidants in normal physiological functions and human disease. Int J Biochem Cell Biol.

[14] Yoshihito I, Silverberg Donald (2012). Anemia Caused by Oxidative Stress. Anemia.

[15] Winterboun CC (2008). Reconciling the chemistry and biology of reactive oxygen species. Nature Chem Biol.

[16] Halliwell B, Gutteridge JM (2007). Free radicals in biology and medicine.

[17] Junior EB, da Silva DG, Torres Lde S, de Almeida EA, Cancado RD, Chiattone C, Bonini-Domingos CR (2012). Oxidative stress and antioxidant capacity in sickle cell anaemia patients receiving different treatments and medications for different periods of time. Annals Hematol.

[18] Walter PB, Fung EB, Killilea DW, Jiang Q, Hudes M, Madden J, Porter J, Evans P, Vichinsky E, Harmatz P (2006). Oxidative stress and inflammation in iron overloaded patients with β-thalassaemia or sickle cell disease. Br J haematol.

[19] Belcher JD, Beckman JD, Balla G, Balla J, Vercellotti G (2010). Heme degradation and vascular injury. Antioxidant Redox Signal.

[20] Niehaus WG, Samuelsson B (1968). Formation of Malonaldehyde from Phospholipid Arachidonate during Microsomal Lipid Peroxidation. Eur J Biochem.

[21] Mesquita CS, Oliveira R, Bento F, Geraldo D, Rodrigues JV, Marcos JC (2014). Simplified 2,4 -dinitrophenylhydrazine spectrophotometric assay for quantification of carbonyls in oxidized proteins. Anal Biochem.

[22] Garrat DC (1964). The Quantitative analysis of drugs.

[23] Sedlak J, Lindsay RH (1968). Estimation of Total Protein-Bound, and Nonprotein Sulfhydryl Groups in Tissue with Ellman's Reagent. Anal Biochem.

[24] Sun M, Zigma S (1978). An Improved Spectrophotometer Assay of Superoxide Dismutase Based On Epinephrine Antioxidation. Anal Biochem.

[25] Awoyemi OM, Bawa-Allah KA, Otitoloju AA (2014). Accumulation and Anti-oxidant Enzymes as Biomarkers of Heavy Metal Exposure in Clarias gariepinus and Oreochromis niloticus. Appli Ecol Env Sci.

[26] Pari L, Latha M (2004). Protective role of Scoparia dulcis plant extract on brain antioxidant status and lipidperoxidation in STZ diabetic male Wistar rats. BMC Compl Alt Med.

[27] Iwalokun BA, Iwalokun SO, Hodonou SO, Aina AO, Agomo PU (2011). Serum levels of leptin in Nigerian patients with sickle cell anaemia. BMC Blood disorders.

[28] Rogers SC, Ross JG, d'Avignon A, Gibbons LB, Gazit V, Hassan MN, McLaughlin D, Griffin S, Neumayr T, Debaun M, DeBaun MR, Doctor A (2013). Sickle hemoglobin disturbs normal coupling among erythrocyte O2 content, glycolysis, and antioxidant capacity. Blood.

[29] Wood K, Hebbel R, Granger D (2005). Endothelial cell NADPH oxidase mediates the cerebral microvascular dysfunction in sickle cell transgenic mice. FASEB J.

[30] Landburg PP, Teerlink T, Biemond BJ, Brandjes DP, Muskiet FA, Duits AJ, Schnog JB (2010). Plasma asymmetric dimethylarginine concentrations in sickle cell disease are related to the hemolytic phenotype. Blood Cells Mol Dis.

[31] Giera M, Lingeman H, Niessen WM (2012). Recent advancements in the LC- and GC-based analysis of malondialdehyde (MDA): A Brief overview. Chromatographia.

[32] Titus J, Chari S, Gupta M, Parekh N (2004). Pro-oxidant and anti-oxidant status in patients of sickle cell anaemia. Indian J Clin Biochem.

[33] Pandey KB, Mishra N, Rizvi SI (2010). Protein oxidation biomarkers in plasma of type 2 diabetic patients. Clin Biochem.

[34] Chirico EN, Pialoux V (2012). Role of oxidative stress in the pathogenesis of sickle cell disease. Int Union Biochem Mol Biol Life.

[35] Alsultan AI, Seif MA, Amin TT, Naboli M, Alsuliman AM (2010). Relationship between oxidative stress, ferritin and insulin resistance in sickle cell disease. Eur Rev Med Pharmacol Sci.

[36] Dasgupta T, Hebbel RP, Kaul DK (2006). Protective effect of arginine on oxidative stress in transgenic sickle mouse models. Free Radic Biol Med.

[37] Gizi A, Papassotiriou I, Apostolakou F, Lazaropoulou C, Papastamataki M, Kanavaki I, Kalotychou V, Goussetis E, Kattamis A, Rombos I, Kanavakis E (2011). Assessment of oxidative stress in patients with sickle cell disease: the glutathione system and the oxidant-antioxidant status. Blood Cells Mol Dis.

[38] Manfredini V, Lazzaretti LL, Griebeler IH, Santin AP, Brandao VD, Wagner S, Castro SM, Peralba MC, Benfato MS (2008). Blood antioxidant parameters in sickle cell anemia patients in steady state. JAMA.

[39] Silva DG, Belini JE, Torres LS, Ricci JO, Lobo CC, Bonini-Domingos CR, de Almeida EA (2011). Relationship between oxidative stress, glutathione S- transferase polymorphisms and hydroxyurea treatment in sickle cell anemia. Blood Cells Mol Dis.

[40] Adelekan DA, Thurnham DI, Adekile AD (1989). Reduced antioxidant capacity in paediatric patients with homozygous sickle cell disease. Eur J Clin Nutr.

[41] Cole SP, Deeley RG (2006). Transport of glutathione and glutathione conjugates by MRP1. Trends Pharmacol Sci.

[42] Reid M, Jahoor F (2001). Glutathione in disease. Curr Opin Clin Nutr Metab Care.

[43] Morris C, Suh JH, Hagar W, Larkin S, Bland DA, Steinberg MH, Vichinsky EP, Shigenaga M, Ames B, Kuypers FA, Klings ES (2008). Erythrocyte glutamine depletion, altered redox environment, and pulmonary hypertension in sickle cell disease. Blood.

[44] Tatum VL, Chow CK (1996). Antioxidant status and susceptibility of sickle erythrocytes to oxidative and osmotic stress. Free Radic Biol Med.

[45] Pulaski L, Jedlitschky G, Leier I, Buchholz U, Keppler D (1996). Identification of the multidrug-resistance protein (MRP) as the glutathione-S-conjugate export pump of erythrocytes. Eur J Biochem.

